# Physician perspective on the implementation of risk mitigation strategies when prescribing opioid medications: a qualitative analysis

**DOI:** 10.1186/s12913-023-10136-z

**Published:** 2023-10-31

**Authors:** Vaishnavi Tata, Randa Al Saadi, Sang Kyu Cho, Tyler J. Varisco, Matthew Wanat, J. Douglas Thornton

**Affiliations:** 1 Department of Pharmaceutical Health Outcomes and Policy, University of Houston College of Pharmacy, 4349 Martin Luther King Jr. Blvd, Houston, TX 77204 USA; 2 Prescription Drug Misuse and Education Research (PREMIER) Center, University of Houston College of Pharmacy, Houston, TX 77204 USA; 3 College of Pharmacy, Department of Pharmacy Practice and Translational Research, University of Houston College of Pharmacy, Houston, TX 77204 USA

**Keywords:** Risk Mitigation Strategy, Implementation science, Qualitative analysis, Thematic analysis, Opioid risk mitigation

## Abstract

**Objective:**

To understand the physician perspective on the barriers and facilitators of implementing nine different opioid risk mitigation strategies (RMS) when prescribing opioid medications.

**Methods:**

We created and dispersed a cross-sectional online survey through the Qualtrics© data collection platform among a nationwide sample of physicians licensed to practice medicine in the United States who have prescribed an opioid medication within the past year. The responses were analyzed using a deductive thematic analysis approach based on the Consolidated Framework for Implementation Research (CFIR) to ensure a holistic approach to identifying the barriers and facilitators for each RMS assessed. In concordance with this method, the themes and codes for the thematic analysis were defined prior to the analysis. The five domains within the CFIR were used as themes and the 39 nested constructs were treated as the codes. Two members of the research team independently coded the transcripts and discussed points of disagreement until consensus was reached. All analyses were conducted in ATLAS.ti© V7.

**Results:**

The completion rate for this survey was 85.1% with 273 participant responses eligible for analysis. Intercoder reliability was calculated to be 82%. Deductive thematic analysis yielded 2,077 descriptions of factors affecting implementation of the nine RMS. The most salient code across all RMS was Knowledge and Beliefs about the Intervention, which refers to individuals’ attitudes towards and value placed on the intervention. Patient Needs and Resources, a code referring to the extent to which patient needs are known and prioritized by the organization, also emerged as a salient code. The physicians agreed that the patient perspective on the issue is vital to the uptake of each of the RMS.

**Conclusions:**

This deductive thematic analysis identified key points for actionable intervention across the nine RMS assessed and established the importance of patient concordance with physicians when deciding on a course of treatment.

**Supplementary Information:**

The online version contains supplementary material available at 10.1186/s12913-023-10136-z.

## Introduction

While the United States grapples with the ongoing opioid epidemic, healthcare practitioners face the continued challenge of treating patients experiencing physical pain. The increase in opioid-related overdose deaths has prompted legislation and policy changes at the federal, state, and local levels – all with the intent to reduce opioid-related morbidity and mortality [1–4]. Within the last ten years, these policies have resulted in a decrease in the overall national opioid prescribing and dispensing rates [5]. However, hesitancy to prescribe opioid medications can be associated with undertreated pain in certain circumstances – particularly where patients with chronic pain are involved [1, 6–8]. Overprescribing of opioid medications has long been a contributor to the opioid crisis and recent years have shown a decrease in the overall number of opioid prescriptions [9, 10]. However, it is imperative that we scrutinize where the decline is [6]. If the number of opioid prescriptions is decreasing among the population of patients with chronic pain, who are in treatment with high-dose opioids, then the risk of harm increases as these patients are not being tapered off appropriately [6]. Additionally, even if legislation proposing stringent limits on the day supply and refill eligibility of these medications does not apply to those with chronic pain, the lack of availability and accessibility of pain management specialists increases the likelihood of undiagnosed, misdiagnosed, and inadequately treated pain [6, 11]. Therefore, the answer to the opioid crisis cannot be to indiscriminately cease prescribing opioids. Instead, more caution is required when assessing the need for an opioid medication as well as what steps should be taken to adequately protect the patient when recommending opioid therapy.

Recognizing the need for strategies to reduce the risks associated with opioid medications, the Centers for Disease Control and Prevention (CDC) released a guideline for prescribing opioids in 2016 that outlined a list of risk mitigation strategies (RMS) to prevent opioid misuse, opioid use disorder (OUD), and overdose [12]. The first two recommendations instructed physicians to consider nonpharmacologic and nonopioid therapy for patients with chronic pain. The remaining nine recommendations of the publication details strategies to implement when initiating opioid therapy with the goal of mitigating the risk of OUD and other adverse outcomes. These recommendations include management of the dosing schedule, patient education regarding the treatment plan, screening patients for risk of OUD, reviewing the prescription drug monitoring program (PDMP), preferentially prescribing immediate-release opioids rather than extended-release for new patients with acute pain, and a schedule for following up with patients currently prescribed opioids. Each of the eleven recommendations were evaluated using the Grading of Recommendations Assessment, Development, and Evaluation (GRADE) method to contextualize the quality of evidence and strength each recommendation [13]. This system categorizes each recommendation into one of four types depending on the robustness of the research supporting it [14]. Type 1 represents a high level of confidence in the recommendation because the research supporting it consists of well-designed randomized clinical trials or overwhelming evidence from observational studies. In contrast, type 4 categorization indicates low confidence in the guideline due to insufficient evidence that the estimated effect of the guideline is approximately equal to the true effect [14]. At the time of the 2016 publication, the CDC Guidelines reported that several recommendations did not have sufficient research to assign a GRADE category. Among those that did have sufficient research, all reported a GRADE category of either type 3 or 4 – indicating a pressing need for studies evaluating their effectiveness.

In 2020, the Agency for Healthcare Research and Quality (AHRQ) published a systematic review that provided an update on the strength of evidence for the recommendations from the 2016 CDC publication [15]. This review included 115 randomized controlled trials, 40 observational studies, and seven studies of diagnostic accuracy of opioid risk prediction instruments with the goal of assessing the comparative effectiveness of nonopioid alternatives as well as the effective of different RMS, their current level of uptake, and the strength of evidence for each finding [15]. This report found moderate evidence for prescribing nonopioid alternatives when managing pain, since they performed just as well as opioid medications in the short-term. However, the 2016 guideline recommends nonopioid therapy for patients with long-term chronic pain and the 2020 AHRQ publication did highlight that there has yet to be a long-term study assessing the comparative effectiveness of nonopioid therapy. When it comes to the evidence for effectiveness of RMS when prescribing opioids, the evidence is further lacking. For example, in the case of screening for risk of OUD, studies reported highly inconsistent estimates of diagnostic accuracy, methodological limitations, and little to no assessment of other risk prediction tools. This systematic review also did not find any studies that assessed the effectiveness of risk prediction instruments, treatment management plans, prescription monitoring program review, urine drug screening, and several other RMS on the risk of overdose or misuse.

The CDC updated their 2016 guidelines in their 2022 publication, *CDC Clinical Practice Guideline for Prescribing Opioids for Pain — United States, 2022* [16]. Some key changes in the updated guidelines include: recognizing the need to include a more collaborative decision-making model with input from behavioral health specialists, delineating which recommendations are appropriate for opioid-naïve patients and those that are already receiving opioids, more specific information on when an adjustment of the dosing schedule is needed, and guidance on methods for tapering and discontinuation schedules. While the case for nonopioid and nonpharmacologic therapies has strengthened since the 2016 publication, the same cannot be said about the RMS recommendations when prescribing an opioid medication. The recommendations for mitigating opioid-related harm in the update still have an evidence type categorization of three or four – indicating that there are either insufficient number of studies evaluating their effectiveness or that the studies conducted have serious limitations that prevent their conclusions from being generalized to the population [13, 14]. This could be due to an implementation gap. The studies evaluated in the AHRQ and CDC publications are largely focused on improvements in clinical outcomes such as the patient’s pain and function. While this information is imperative in determining the success of an RMS, it neglects to assess the physician perspective on the implementation of each RMS. If the implementation of a given RMS is infeasible due to barriers from the physician’s perspective, it could lead to either low or inconsistent adoption of the RMS into clinical practice.

The research on risk mitigation when prescribing opioid medications from the physician perspective is sparse, but emerging. One study conducted a series of qualitative panel discussions with 18 national experts to assess their opinions on the CDC Guidelines for opioid prescribing and solicit their recommendations for safe practices [17]. While this study did address the gap in literature concerning the physician perspective, the scope remains too wide. There were no questions regarding specific RMS. Instead, the semi-structured interviews elicited general suggestions for the safe prescribing of opioid medications. Another study addressed the lack of specificity in addressing the actionable points of the guidelines and interviewed nine physicians about the details of implementing them into practice [18]. While this study did garner important information into how physicians could implement the RMS into practice, the sample size is still limited and homogenous in geographic location. It sampled all of the participating physicians from two New York City boroughs – an area that has a high access to healthcare services, which does not hold true for a large portion of patients who are prescribed opioid medications. Lastly, a systematic review on provider perceptions regarding opioid prescribing and substance use treatment policies showed that some RMS are more heavily studied than others (i.e., Reviewing the PDMP Prior to Prescribing an Opioid); however, the studies on these RMS to date have focused on access and realization of access rather than their actual utilization within clinical practice [19].

In order for RMS to be as effective as possible in mitigating the risk associated with opioid medications, there is a critical need to understand the challenges that physicians face when implementing them into clinical practice. In this study, we focused on nine, specific RMS: Documentation of Treatment Goals, Screening for Risk of an OUD, Limiting the Day Supply of an Opioid Prescription, Limiting the Dosage of an Opioid Prescription, Reviewing the PDMP Prior to Prescribing an Opioid Medication, Limiting Concurrent Benzodiazepine Prescriptions, Urine Drug Screening, and Use of Referral Services. The objective of this study was to understand the physician perspective on these nine RMS, adopted from the CDC’s guideline on opioid prescribing, and experience with them in clinical practice.

## Methods

### Study design, sample, and recruitment

In this study, we developed a cross-sectional, online survey among a nationwide panel of physicians to assess their perspective on nine different RMS. The sample was identified by Qualtrics®, a data collection and management company that provides a platform for online surveys. Qualtrics® recruits participants from various sources, including website intercept recruitment, member referrals, targeted email lists, gaming sites, customer loyalty web portals, permission-based networks, and social media. Consumer panel members’ names, addresses, and dates of birth are typically validated by Qualtrics via third-party verification measures prior to their joining a panel. Business-to-business participants are subject to additional quality control measures such as LinkedIn matching, phone calls to the participant’s place of business, and other third-party verification methods (TrueSample, RelevantID, Verity, etc.) [20]. As a result of their diverse recruitment procedure, some participants may have been offered an incentive in the form of SkyMiles, or points towards retail purchases, on behalf of Qualtrics®. No direct incentive was offered by the research team themselves.

Participants were provided with an anonymous link to the survey, which started with the informed consent. If participants consented to be part of the study, they were then taken to the screening criteria. Only physicians who were currently licensed to practice in the United States and had prescribed an opioid medication within the past year were included in the study. Any respondents who either indicated that they did not wish to participate in the study or did not meet the screening criteria were excluded. Respondents were also excluded if they did not complete the survey instrument in its entirety. The data in this study was collected in January 2022. This study was reviewed and approved by the University of Houston Institutional Review Board.

### RMS included and survey structure

The nine different RMS included in this survey were: Documentation of Treatment Goals (RMS1), Screening for Risk of Opioid Use Disorder (RMS2), Limiting the Day Supply of an Opioid Prescription (RMS3), Limiting the Dosage of an Opioid Prescription (RMS4), Reviewing the PDMP Prior to Prescribing an Opioid Medication (RMS5), Limiting Concurrent Benzodiazepine Prescriptions (RMS6), Urine Drug Screening (RMS7), Safe Drug Disposal (RMS8), and the Use of Referral Services (RMS9). These nine were determined by the recommendations made in the CDC’s 2016 publication for guidelines when prescribing opioid medications. A brief description of each one was provided to the participants and these descriptions can be found in Table 1 of the Tables and Figures section.

**Table 1:** Demographic Characteristics of Nationwide Physician Sample

		Physicians n = 273	%
SEX			
	Male	186	68.13%
	Female	87	31.87%
AGE			
	26 to 35	14	5.13%
	36 to 45	55	20.15%
	46 to 55	97	35.53%
	56 to 65	78	28.57%
	66+	29	10.62%
RACE			
	White	184	67.40%
	Black or African American	7	2.56%
	Hispanic or Latino	8	2.93%
	Asian or Other Pacific Islander	70	25.64%
	Other^1^	4	1.47%
Have you ever received an opioid medication?			
	Yes	157	57.51%
	No	116	42.49%
PRACTICE SPECIALTY			
	General Practice	183	67.03%
	Anesthesiology, Pain Medicine, and Rehabilitation	41	15.02%
	Other^2^	42	15.38%
	Unknown	7	2.56%

^1^ Refers to Native American, Unknown, or Preferred not to Say

^2^ Refers to Addiction Medicine, Dermatology, Emergency Medicine, Gastroenterology, Geriatrics, Hematology, Oncology, Neurology, Obstetrics and Gynecology, Orthopedics, Palliative Medicine, Pediatrics, Psychiatry, Rheumatology, or Unknown

Eligible participants were asked to review a brief description for each RMS and subsequently asked to rate their level of comfort, perceived effectiveness, and ease of implementation for each. To rate these constructs, participants were presented with a seven-point Likert scale with lower numbers representing lower levels of each construct. They were then asked an open-ended question to provide the rationale for their rating [21]. This manuscript focuses on the qualitative thematic analysis of the open-ended question responses provided by the physicians; however, given that the quantitative results to shed more light into the qualitative responses, descriptive averages of each construct were totaled. The open-ended responses for each RMS were compiled into a separate transcript, resulting in a total of nine transcripts, and used as the unit of analysis. Participant demographics such as age, sex, race, and practice specialty information were also collected. As part of the demographic questions, participants were also asked if they had ever been prescribed an opioid medication in the past. The survey administered to the participants can be found in the supplemental materials section of this paper.

### Data analysis

The scores for each construct physicians rated on a seven-point Likert scale were developed by averaging all physician scores for the same construct. From there, the construct averages were averaged for a total aggregate feasibility score. These descriptive averages are intended to give context to the qualitative responses, but a full quantitative analysis is currently underway to be published at a later date.

The open-ended responses were analyzed using a deductive thematic analysis approach. Thematic analysis is a qualitative, descriptive method used to identify and analyze repeating patterns within a set of data [22–25]. While thematic analysis is primarily an inductive method of analysis, it has been applied in deductive research when a theoretical framework (COM-B model, transtheoretical model, etc.) is used to establish an *a priori* list of themes and codes [26–28]. In this study, the Consolidated Framework for Implementation Research (CFIR) was used [21]. The CFIR framework provides a repository of standardized implementation-related constructs that can be applied across the spectrum of implementation research. It consists of 39 constructs nested across five major domains, all of which interact to influence implementation and implementation effectiveness [21, 29]. It has been used extensively in healthcare-related research to identify actionable points of intervention to increase the uptake and implementation of a health policy or process [30–32].

In accordance with the deductive thematic analysis approach, the codebook was developed prior to coding based on the CFIR [33]. The five domains within the CFIR framework (Outer Setting, Inner Setting, Characteristics of Individuals, Process, and Intervention Characteristics) were used as themes in the analysis. Similarly, the 39 constructs provided by the CFIR were declared codes for the analysis and nested under the corresponding themes.

To establish the dependability and reliability of the analysis process, two independent coders conducted the thematic analysis separately with the agreed upon codebook and iteratively discussed their coding until consensus was reached [28]. Both coders maintained an audit trail of their codes and notes [28].

1. *Data Familiarization*: Each member read the nine documents without coding, only to review the descriptions and responses to each RMS.
2. *Coding*: The nine documents were uploaded into ATLAS.ti© v7 [34] and two separate copy bundles were created for each of the coders from the same hermeneutic unit file. Each coder used the established CFIR codebook to independently code all nine transcripts.
3. *Merging*: Once independent coding was complete, the two copy bundles were merged to create one hermeneutic unit with unified codes. Intercoder reliability was assessed via percent agreement between the two coded files within the ATLAS.ti© v7 [34] software.
4. *Arbitration and Code Management*: During this phase, any quotes that were coded differently were discussed and a solution was agreed upon by the two coders. Any codes that were not attached to quotes within the transcripts were removed from the final codebook.
5. *Producing the Report*: The final report consisted of the coded, merged, and reconciled nine transcripts along with the codebook and accompanying thematic map.

## Results

Prior to applying the exclusion criteria, 321 physician responses were collected. After exclusion, 273 participant responses eligible for analysis, resulting in completion rate of 85.1%. All eligible participants responded to the open-ended question asking them to give the rationale behind their rating. Participant demographic information can be found in Table 2. The sample primarily consisted of white (n = 184, 67.4%) males (n = 186, 68.1%) currently working in general practice (n = 183, 67.0%) that had personally received an opioid medication in the past (n = 157, 57.5%). The descriptive averages for each construct score and the aggregate feasibility score for each RMS can be found in Table 3. RMS5 was rated the highest across all constructs and ultimately resulted in the highest aggregate feasibility score as well. RMS9 had the lowest aggregate feasibility score, but only had the lowest ease of implementation score of the RMS. It still had higher comfort and ease of implementation scores.

**Table 2:** Nine Risk Mitigation Strategies Assessed

Risk Mitigation Strategy [60]	Short Description
Documentation of Treatment Goals (RMS1)	The CDC recommends that before starting opioid therapy for chronic pain, clinicians should establish treatment goals with all patients, including realistic goals for pain and function. Clinicians should also consider how to discontinue opioid therapy if benefits do not outweigh risks. These treatment goals should be documented and agreed between patient and physician.
Screening for Risk of Opioid Use Disorder (RMS2)	The concern for addiction risk is shared by physicians, patients, and their families. Some patients with chronic pain may suffer from a prior history of addiction. Alternatively, even patients without any history of substance use issues may go on to develop aberrant drug-related behaviors (ADRB). Therefore, it is important to screen patients for their risk of developing an opioid use disorder (OUD) before initiating opioid therapy. In order to screen patients for their risk of developing an OUD, there are many different tools. Some examples are: (1) Opioid Risk Tool (ORT) – This is a 5-question screening tool designed for use in adults to assess for the risk for opioid abuse or ADRB. (2) Screener and Opioid Assessment for Patients with Pain-Revised (SOAPP®-R) – This is a 24-question screening tool that is designed to predict ADRB prior to the initiation of long-term opioid therapy.
Limiting the Day Supply of an Opioid Prescription (RMS3)	The CDC recognizes that long-term opioid use often begins with the treatment of acute pain. Therefore, in order to prevent long-term reliance on opioid medications, they recommend that clinicians limit the day supply of opioid prescriptions to three days or less for the treatment of acute pain. They also state that prescriptions for more than seven days will rarely be needed and should be avoided.
Limiting the Dosage of an Opioid Prescription (RMS4)	One way to mitigate the potential for dependence is by prescribing lower dosages of opioids. Higher doses of opioids do not always translate to more pain relief due to tolerance. Therefore, placing a maximum dosage cap may prevent opioid misuse.
Reviewing the PDMP Prior to Prescribing an Opioid Medication (RMS5)	Prescription Drug Monitoring Programs (PDMPs) are state-maintained databases that keep track of controlled substance prescribing and dispensing within the state and other partnered states using the same software platform. They can be used to identify a patient’s history of controlled substances, as well as concomitant use of controlled substances.
Limiting Concurrent Benzodiazepine Prescriptions (RMS6)	Patients who have taken benzodiazepine and opioid medication concurrently have been known to experience adverse events ranging from respiratory depression to overdose. Recognizing the risk of co-prescribing, in 2016 the US Food and Drug Administration issued a warning to patients and clinicians about the potential risks of combined opioid and benzodiazepine use. However, the panel that wrote the guidelines noted that there may be clinically appropriate situations where the drugs may be used together, giving the example of a person taking a stable low dose of a benzodiazepine who experiences acute pain.
Urine Drug Screening (RMS7)	Urine drug testing, also known as urine drug screening, is a painless test that analyzes your urine for the presence of certain prescription medications. The CDC recommends urine drug screening both before the initiation of opioid therapy and at least annually during opioid therapy.
Safe Drug Disposal (RMS8)	One way to reduce the number of opioid-related overdoses and deaths is to ensure the proper disposal of opioid medications. This can be done through a variety of ways. The Drug Enforcement Administration (DEA) sponsors National Drug Take Back days, where central locations are set up for residents to drop off their unused or leftover medications to be disposed of safely. Another alternative is Single Use Disposal Systems (SUDS). These systems allow for patients to safely dispose of their medications from home. Physicians can play an important role in ensuring that patients are aware of the various safe disposal options available to them.
Referral Services (RMS9)	Physicians use a variety of tools (PDMP review, urine drug testing, OUD Screening) to assess the potential of opioid dependency among their patients. If a patient is thought to be misusing or developing a dependency on their opioid medications, physicians can refer their patients to treatment. More specifically, the FDA recommends Medication Assisted Treatment (MAT) to treat OUD.

**Table 3:** Codebook Developed from the Consolidated Framework for Implementation Research Framework [21]

Theme		
	Code	Short Description
INTERVENTION CHARACTERISTICS		
	Cost	Costs of the intervention and costs associated with implementing the intervention including investment, supply, and opportunity costs.
	Complexity	Perceived difficulty of implementation, reflected by duration, scope, radicalness, disruptiveness, centrality, and intricacy and number of steps required to implement.
	Evidence Strength and Quality	Stakeholders’ perceptions of the quality and validity of evidence supporting the belief that the intervention will have desired outcomes.
	Adaptability	The degree to which an intervention can be adapted, tailored, refined, or reinvented to meet local needs.
OUTER SETTING		
	Peer Pressure	Mimetic or competitive pressure to implement; typically because most or other peer or competing organizations have already implemented or are in a bid for a competitive edge.
	External Policies and Incentives	A broad construct that includes external strategies to spread interventions, including policy and regulations (governmental or other central entity), external mandates, recommendations and guidelines, pay-for-performance, collaboratives, and public or benchmark reporting.
	Patient Needs and Resources	The extent to which patient needs, as well as barriers and facilitators to meet those needs, are accurately known and prioritized by the organization.
INNER SETTING		
	Culture	Norms, values, and basic assumptions of a given organization.
	Implementation Climate	The absorptive capacity for change, shared receptivity of involved individuals to an intervention, and the extent to which use of that intervention will be rewarded, supported, and expected within their organization.
	Relative Priority	Individuals’ shared perception of the importance of the implementation within the organization.
	Structural Characteristics	The social architecture, age, maturity, and size of an organization.
PROCESS		
	Opinion Leaders	Individuals in an organization who have formal or informal influence on the attitudes and beliefs of their colleagues with respect to implementing the intervention.
	Reflecting and Evaluating	Quantitative and qualitative feedback about the progress and quality of implementation accompanied with regular personal and team debriefing about progress and experience.
	Intervention Participants	Recipients of the intervention and their level of engagement and involvement within the intervention.
	Champions	Individuals who dedicate themselves to supporting, marketing, and ‘driving through’ an implementation, overcoming indifference or resistance that the intervention may provoke in an organization.
CHARACTERISTICS OF INDIVIDUALS		
	Individual Stage of Change	Characterization of the phase an individual is in, as he or she progresses toward skilled, enthusiastic, and sustained use of the intervention.
	Self-Efficacy	Individual belief in their own capability to execute actions to achieve implementation goals.
	Knowledge and Beliefs about the Intervention	Individuals’ attitudes toward and value placed on the intervention as well as familiarity with facts, truths, and principles related to the intervention.

*Note: All themes, codes, and definitions are directly from the CFIR

**Fig. 1:**
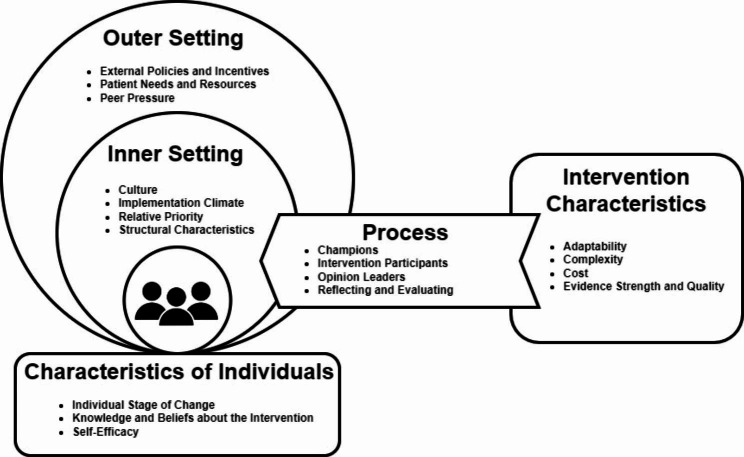
Thematic Map

After merging both independently coded transcripts, intercoder reliability was calculated within the ATLAS.ti© software and found to be 82%. All differing quotes were discussed by the two coders and arbitrated by another research team member until 100% agreement on the quotations used was achieved. Any codes without any quotations attached to them were removed from the final codebook. The final codebook had 18 codes nested across five themes from the CFIR framework. This can be found in Table 4. The relationship between the themes and their respective codes is visually represented in the thematic map in Fig. 1 [35]. Deductive thematic analysis yielded 2,077 descriptions of factors affecting implementation of the nine RMS. The most salient codes across all nine RMS were Knowledge and Beliefs about the Intervention and Patient Needs and Resources. Detailed results regarding each of the five themes and their nested constructs are below.

**Table 4:** Average physician responses to each construct assessed and averaged acceptability score

Risk Mitigation Strategy	Comfort	Effectiveness	Ease of Implementation	Aggregate Feasibility Score
Documentation of Treatment Goals	5.75	4.90	4.44	5.03
Screening for Risk of Opioid Use Disorder	5.21	4.81	4.39	4.80
Limiting the Day Supply of an Opioid Prescription	5.51	5.18	4.97	5.22
Limiting the Dosage of an Opioid Prescription	5.56	5.14	5.07	5.26
Reviewing the PDMP Prior to Prescribing an Opioid Medication	6.20	5.82	5.54	5.85
Limiting Concurrent Benzodiazepine Prescriptions	5.62	5.16	4.85	5.21
Urine Drug Screening	5.64	5.32	4.90	5.28
Safe Drug Disposal	5.58	4.73	4.70	5.00
Referral Services	5.12	4.86	3.94	4.64

### Intervention characteristics

Within the theme of intervention characteristics, there were four codes – Cost, Complexity, Evidence Strength and Quality, and Adaptability. RMS7 resulted in more concerns over cost than any other RMS. Physicians seemed to agree that RMS7 is a simple and straightforward test to evaluate whether a patient is on any other substances or if a patient is taking opioid medications instead of diverting them. However, they did express that the cost to the patient may be prohibitive and the information that RMS7 analysis provides may not be worth the cost to the patient.

Although getting a urine drug sample can be easy, implementation could be difficult as if the patient has no insurance or high deductibles, they might complain of the costs of these tests. Also, sometimes the results can be false positives/negatives which require that a sample has to be sent for a more comprehensive analysis and that takes time and again more cost. What you do in the meantime while waiting for the result is problematic.

When asked about the utility of validated screening tools in the assessment for risk of OUD, physicians indicated that although screening tools may be easy to implement, they would not yield meaningful results as patients would be susceptible to social desirability bias due to self-reporting. This finding is echoed in the CDC guidelines as well as the AHRQ systematic review. Within the CFIR, this compromises the Evidence Strength and Quality of the intervention.

I don’t use these tools as often as I should. The questionnaires are easy to use and can be helpful as a screening tool. However, I have found that they have not been very predictive, and my suspicion is patients do not honestly answer the questions.

“I’m comfortable with this, as I use at least the ORT [Opioid Risk Tool] in patients who come in with chronic pain and are seeking a new provider. Since the patients in question answer the screening tools, the patient might feel it is better to answer what they think is the answer the provider wants and may give them a higher chance of getting the medication vs. what they actually feel or believe. The implementation is fairly easy but cannot agree that the answers themselves would be.”

### Outer setting

The three codes nested under the theme of outer setting are Peer Pressure, External Policies and Incentives, and Patient Needs and Resources. Patient Needs and Resources emerged as one of the most salient themes in this analysis since it shaped whether or not physicians even reported carrying out a given intervention. In the case of referral services, physicians reported a large discrepancy between patient needs and resources available. From the physician perspective, referral services were needed for the majority of patients, however many patients may not buy in to them. Even for patients who were interested in seeking these services, the lack of availability of them makes utilization and treatment difficult.

We are aggressive about directing patients to these resources; however, patients often experience socioeconomic barriers to using them.

I tried to get a patient into [Medication Assisted Treatment (MAT)] and the wait was for three months. Until we can get more doctors to [prescribe] MAT such as suboxone, buprenorphine, etc.- this will be challenging.

There are limited resources, and it also depends on patient buy-in.

Physicians can make the referral, but patients may be resistant.

The same sentiment was echoed in the case of safe drug disposal. Medication disposal is an important part in preventing prescription drug misuse and diversion, but physicians indicated that it is an inconvenient process to patients, citing reasons such as lack of disposal sites, infrequent and inconvenient locations of medication take back events, and increased burden to patients. Physicians claimed that medication disposal would be an effective RMS but are likely poorly implemented in most communities due to their lack of adaptability to meet patient needs.

The Take Back Days are a farce-they are too infrequent and so poorly advertised, that when I went into my local Precinct a few years ago to ask about the next day’s collection, none of the officers were even aware it was going to take place, or that it had ever been done there in the past. On the other hand, local pharmacies that have a lock box where drugs can be deposited at any time are useful.

Patients perceive having to take medications somewhere as an inconvenience.

These events are few and far between. I am almost never aware of them until I hear about them on the radio or some other random way. I don’t feel physicians are notified about it by the DEA or any other agency.

Physicians indicated that while limiting the day supply of an opioid prescription would be effective in mitigating the risk of dependence, they anticipate that patients would be very resistant to this RMS. Additionally, they brought up the concern that while this RMS is more appropriate for acute pain, it would lead to inadequate pain management for those with chronic pain or more severe injury.

I feel that in theory there are patients where you could use this strategy, but some would be very opposed to this or wouldn’t understand why they would only be able to obtain such a short duration if they patient genuinely had pain issues that needed to be addressed and might leave the patient being less satisfied with care than they might be otherwise.

I like the idea of limiting the acute supply to 3 days but limiting overall opioids supply to seven days will be resisted by some patients. I agree that longer than seven days is rarely needed, but I do not think it is never needed. Guidance as to when seven days should be allowed would be helpful.

The same concern was raised over limiting the dosage or instantiating a maximum dosage for opioid medications. Physicians raised concerns that a hard cap on opioid dosage may lead to inadequate management for patients with more severe pain.

I think that this strategy would be easier to implement overall for patients but if a certain dosage doesn’t work for patients, it would be hard to tell them you wouldn’t be able to increase it. And as such there are different levels of pain that patients experience as well as different in medication efficacy based on varying factors such as weight, age, sex, previous exposure to pain medications to name a few.

This strategy may be ‘easy’ to implement in that it will likely be interpreted as encouraging a fixed maximum opioid dose, that may neglect the needs of small number of patients. It would be helpful would be include guidance regarding when higher doses are appropriate and how to add additional safety measures to improve clinical outcomes.

Lastly, external policies and mandates regarding the need to review patients in the PDMP prior to prescribing controlled substances has created a unique problem for physicians. Perceptions of the utility and potential of the PDMP as a tool to identify patterns of misuse and diversion were overwhelmingly positive. Physicians in this sample were nearly unanimous in their opinion that the PDMP is an effective tool at the point of care. The primary barriers reported to implementation of regular PDMP review were logistical in nature. Concerns over the time constraint of reviewing every patient were pervasive.

This is helpful but applies another layer of physician interaction with a database that takes the providers time away from patient care.

This mitigation strategy can be very effective but is sometimes time consuming and challenging to implement.

### Inner setting

Inner Setting was the overarching theme that consisted of the codes Culture, Implementation Climate, Relative Priority, and Structural Characteristics. Within this theme, physicians agreed that the ongoing opioid crisis had brought additional attention and scrutiny to prescribing opioid medications. They did report a supportive culture that places a priority on implementing RMS, but did address that the Implementation Climate may not be as supportive for some RMS. For example, in the case of RMS6, physicians indicated that they understood the risks of co-prescribing benzodiazepines and opioids and refrained from doing it themselves. They believe concurrent prescriptions is often the result of multiple prescribers. Mental health comorbidities are prevalent among the substance use population [36–41]. It is not uncommon for comorbid patients to see multiple specialists depending on their comorbidities in addition to their primary care or pain management physician [42]. A lack of collaboration between the multiple providers and a lack of integration of electronic medical records may contribute to the concurrent use of dangerous combination of medications.

More difficult since it is usually another provider, usually mental health, who is prescribing the Benzo[diazepine].

I don’t tend to prescribe benzo[diazepine]s in my practice, however they could easily get from their PCPs.

These factors are considered when prescribing opioids for any patient or on these other medications. Our goal is to minimize dosing and scheduling for these patients and at times consider transferring their care to pain management.

### Process

When it comes to the actual process for implementation of the RMS, the codes prevalent were Opinion Leaders, Reflecting and Evaluating, Intervention Participants, and Champions. Under the code Intervention Participants, physicians indicated that patients may not be in the right frame of mind or may lack the technical knowledge to understand the goals and boundaries set in their initial treatment goals conversation.

[Patients] will basically agree to anything at first. If they don’t meet goals however, frequently they ‘won’t remember’ what the initial goals were or what you said.

It is easy to discuss these things, but most patients are not listening to these goals. They just want the medications and will agree to whatever you say without really understanding it.

While it is true that most patients lack the degree of clinical training and expertise that physicians are equipped with, removing the patient as a key stakeholder within their own treatment plan is not the answer. Patient “buy-in” to their treatment goals and an understanding of what would be required of them is imperative to treatment retention.

### Characteristics of individuals

The most pervasive code, Knowledge and Beliefs About the Intervention, is grouped under the theme of Characteristics of Individuals. A key finding that emerged was a lack of consensus or guidance on what each RMS recommended consisted of. One example of this is how physicians reported establishing treatment goals with their patients. While most physicians in this study reported having conversations with their patients about the goals and general plan for the duration of treatment, wide variation in the method of documentation persists. Lack of consensus on the content and structure of this documentation may compromise the effectiveness of the RMS.

I feel that the documentation is something the majority of physicians and mid-level providers already do, although some may take some shortcuts in doing this. The effectiveness of the strategy depends on the particular provider in question, as some may gloss over it while others give it more serious thought. Implementation would be difficult without some type of template that can scan electronic medical records to look for adherence to the recommendation.

## Discussion

Overall, physicians believed that the RMS evaluated in this study would all contribute to mitigating the risk associated with prescribing opioid medications. However, key stakeholder buy-in and proving the effectiveness of a given intervention does not always translate to meaningful patient outcomes within healthcare settings. In this study, we used the CFIR framework to evaluate what the barriers and facilitators of implementation were from a physician perspective. From this we were able to identify some actionable intervention points to improve the standard and process of care when prescribing opioid medications.

### Reviewing the prescription drug monitoring program prior to prescribing an opioid medication

Prescription Drug Monitoring Programs (PDMPs) are electronic databases that keep track of controlled substance prescription dispensing within a state and within other collaborative states with similar software platforms [43, 44]. Currently, providers in all 50 states, the District of Columbia, Puerto Rico, and Guam have access to a PDMP and many states now mandate that prescribers and pharmacists review a patient’s PDMP profile prior to prescribing or dispensing controlled substance medications [44]. Support for PDMPs in clinical practice is unassailable, however, this enthusiasm for their potential in mitigating misuse and diversion has yet to translate to actual use of them.

In this study, we found that physicians cited time constraints as the primary reason for their lack of querying patients in the PDMP. This finding is consistent with findings from prior studies assessing the adaptability of the PDMP into clinical practice [45–49]. To increase the rate of utilization of PDMPs in clinical practice, it is imperative that we enable physicians to assign delegates (qualified members of their medical practice) that can query patients on their behalf. Another way to increase PDMP uptake would be to encourage the integration of PDMPs with electronic health records. This would allow for easier access to the data for healthcare providers and allow data sharing between clinicians prescribing and dispensing these medications. Having multiple healthcare professionals with access to the patient’s medication information introduces more checkpoints where the potential for adverse reactions or signs of misuse can be identified and intervened upon. Several states have already introduced some level of integration between their PDMP and EHRs with favorable results [50, 51]. In our current study, physicians also call for a PDMP at the national level instead of by individual state allowing the creation of data sharing agreements.

### Safe drug disposal

Removing unused or leftover medications from the home can reduce the risk of others taking the medication accidentally or misusing the medication intentionally [52, 53]. Previously, disposal methods were limited to three options. For disposal at home, the Food and Drug Administration (FDA) historically recommended flushing unused medication – a stance they have since denounced due to environmental concerns [54]. Alternatively, the Environmental Protection Agency (EPA) recommended removing unused medications from the home by removing the medication from its original container and mixing it with some unwanted substance such as coffee grounds or kitty litter and then disposing in regular household trash [55]. Another option for medication disposal is national Take Back Days, an initiative primarily driven by the Drug Enforcement Administration (DEA) [52]. However, physicians in this study expressed these events to be infrequent, not properly advertised, and often geographically inconvenient for the majority of patients to use as their primary method of medication disposal [52].

A viable solution to the issues presented with traditional drug disposal methods are Single Use Disposal Systems (SUDS) [56, 57]. SUDS allow consumers to safely dispose unused medication and provide an environmentally friendly alternative to flushing medication down the toilet or discarding in the trash [56]. All SUDS can be categorized into one of two groups based on their method of disposal: deactivation or incineration. Deactivation products use a chemical process to denature medications added to the system, rendering them inert. Incineration works by providing patients with a prepaid envelope in which they can ship their unused medications to a central location where the incineration process is handled by experts. Both disposal avenues allow patients to conveniently dispose their medications without leaving their home. Recognizing the potential of SUDS in mitigating the risk of unused medications, several states are making efforts to make them more available to patients through physician offices, pharmacies, and other community-level partners [56–59].

### The patient perspective

Lastly, and perhaps most importantly, physicians expressed the importance of the patient perspective across all RMS in this study. In discussing the documentation of treatment goals, the buy-in and decision-making input from patients was repeatedly described as imperative to the success of treatment. When it comes to screening for risk of OUD, physicians expressed concern over the validity of these tools because patients self-report more favorably, and thus physicians prefer to have a conversation with the patient instead. In both the cases of RMS3 and RMS4, respondents indicated that they would be comfortable using this method to limit misuse, but not at the risk of leaving patients’ pain inadequately managed. For RMS7, the primary concern was cost to the patient. With safe drug disposal and referral services, the lack of availability to patients was paramount to the physicians within this sample.

Ultimately, medicine is a collaborative practice that requires engagement from all key stakeholders – especially patients. The need for the patient perspective on the RMS strategies is imperative to their overall success in mitigating the risks associated with opioid medications. Once the patient perspective is appropriately assessed, the next step in the process will be to identify actionable points of intervention to increase patient-physician concordance on the RMS.

### Limitations

This study does have a few limitations. First, the preliminary nature of the research question and the number of RMS assessed in this study did not allow for detailed assessment of each factor contributing to the overall acceptability of each RMS. The goal of this research study was to gather enough cursory information to narrow down which RMS were most acceptable to both patients and physicians. The information from this study will allow us to design future studies exploring the most acceptable RMS in further detail. Second, in the data collection process, the research team did not specify that information on how many participants were excluded with each screening criterion had to be collected. Therefore, only the total number excluded after all screening criteria had been applied was collected. Third, the qualitative responses collected were from an unstructured, open-ended question asking the participants to explain the reasoning behind their rating. When the survey was designed, this question was added to give some insight into the ratings, but the research team did not anticipate the completeness and robustness of the qualitative responses provided. A qualitative analysis was decided on after reviewing the data collected. As a result, the level of detail regarding each individual rating is limited.

## Conclusion

In this study, we evaluated the physician perspective on nine separate RMS with the intention to identify the factors that facilitate and impede strategy implementation in clinical practice. To the authors’ knowledge, this is the first study to evaluate the physician perspective on these nine RMS. The three predominant factors identified have prompted suggestions of a few interventions that can increase the adoption of the RMS among physicians and subsequently reducing the negative outcomes associated with opioid medications. However, there is still a critical need for more in-depth research into how to effectively combine individual RMS as well as the perspectives of other key stakeholders such as patients.

### Electronic supplementary material

Below is the link to the electronic supplementary material.

Supplementary Material 1

## Data Availability

The raw data for this project consists of the transcripts of qualitative responses from the physicians. This data is not publicly available to the public as it is not allowed by our Institutional Review Board, but it will be made available by the corresponding author on reasonable request.
