# Supplementary material for: Physician perspective on the implementation of risk mitigation strategies when prescribing opioid medications: a qualitative analysis
Source: BMC Health Serv Res. 2023 Oct 31;23:1185. doi: 10.1186/s12913-023-10136-z (PMC10617230; doi:10.1186/s12913-023-10136-z)
Supplement: Supplementary file 1 [file 12913_2023_10136_MOESM1_ESM.docx]

RMS Survey - Physicians

Start of Block: Default Question Block

Q1 **Assessing Determinants of Opioid Risk Mitigation Strategies Among Physicians and Patients**

*Investigator: Dr. J. Douglas Thornton, PharmD, PhD, BCPS*

***Key Information:*** The following focused information is being presented to assist you in understanding the key elements of this study, as well as the basic reasons why you may or may not wish to consider taking part. This section is only a summary; more detailed information, including how to contact the research team for additional information or questions, follows within the remainder of this document under the “Detailed Information” heading.

*What should I know about a research study?*

Taking part in the research is voluntary; whether or not you take part is up to you. You can choose not to take part. You can agree to take part and later change your mind. Your decision will not be held against you. You can ask all the questions you want before you decide and can ask questions at any time during the study. As part of the long-term effort to alleviate the opioid crisis, the Centers for Disease Control and Prevention CDC updated a 2014 systematic review on the effectiveness and risks of opioids to introduce 12 over-arching recommendations for the safe prescribing of opioids as well as several risk mitigation strategies to prevent adverse outcomes. They include documentation of treatment goals, limiting the dosage of an opioid prescription, routine urine drug screening, and more. The effectiveness of opioid risk mitigation strategies to prevent potential abuse and misuse of prescription opioids depends on their feasibility in clinics and acceptability by patients and prescribers.

Against this background, this research project will evaluate determinants of CDC’s recommended opioid risk mitigation strategies. Specifically, this research project will survey prescribers and patients to understand why some opioid risk mitigation strategies are more feasible and acceptable in clinical practice. This research will identify and address barriers associated with each opioid risk mitigation strategy.

You are invited to take part in this study because you are a physician who has prescribed opioids within the past one year. This research is being funded by the Substance Abuse and Mental Health Services Administration (SAMHSA) via the Texas Health and Human Services Commission (THHSC).

In general, your participation in the research involves responding to survey questions regarding nine (9) opioid risk mitigation strategies. Using an online platform, you will rate their acceptability, effectiveness, and ease of implementation for each risk mitigation strategy. There are a total of nine (9) risk mitigation strategies in the survey. You will then be asked to rank order the nine strategies in order of preference.

The surveys are administered to patients and prescribers using an online platform. There is no direct contact between study participants and researchers. There are no foreseeable risks related to the procedures conducted as part of this study. If you experience any negative events due to your participation in this research or if you have any questions prior to, during, or after your participation in this research, please refer to the contact information and instructions at the end of this cover letter.

 ***Detailed Information***: The following is more detailed information about this study, in addition to the information listed above.

*Why is this research being done?*

This research project will evaluate why some opioid risk mitigation strategies are more feasible and acceptable in clinical practice. Understanding the feasibility and acceptability of an opioid risk mitigation strategy will improve the implementation of CDC’s recommended opioid risk mitigation strategies to opioid misuse and abuse, opioid use disorder, and opioid-related hospitalizations and deaths.

*How long will the research last?*

If you agree to participate, we will ask you to participate in a questionnaire. This questionnaire is expected to take you about 20 minutes.

*How many people will be studied?*

We approximate that 400 patients and 265 physicians will be surveyed as part of this research study.

*What happens if I say yes, I want to be in this research?*

Study participants will take an online survey to answer a series of questions related to opioid risk mitigation strategy. Study participants will rate their acceptability and feasibility for each opioid risk mitigation strategy using a number between 1 and 9 and provide the rationale for their rating in a comment box.

*What happens if I do not want to be in this research?*

You can choose not to take part in the research and it will not be held against you. Choosing not to take part will involve no penalty or loss of benefit to which you are otherwise entitled.

*What happens if I say yes, but I change my mind later?*

You can choose not to take part in the research, and it will not be held against you. You can leave the study at any time, and it will not be held against you. If you decide at a later point that you would no longer like to be involved in the research, please email the research team. Any information you have provided up until discontinuation will not be used in the analysis phase and will not be included for publication. 

*Is there any way being in this study could be bad for me?*

There are no foreseeable risks related to the procedures conducted as part of this study. If you choose to take part and undergo a negative event you feel is related to the study, please inform your study team.

*Will being in this study help me in any way?*

There are no known benefits to you from your taking part in this research.

*What happens to the information collected for the research?*

Your taking part in this project is anonymous, and the information you provide cannot be linked to your identity. We may publish the results of this research in academic journals and present the findings at academic conferences. However, unless otherwise detailed in this document, we will keep your name and other identifying information confidential.

*Who can I talk to?*

If you have questions, concerns, or complaints, or think the research has hurt you, you should talk to the research team at the University of Houston’s PREMIER Center. The primary investigator of this research is Dr. Douglas Thornton. He can be reached at jdthornt@central.uh.edu. You can also reach out to the graduate research assistant on this project, Vaishnavi Tata. She can be reached at vtata@central.uh.edu. 

This research has been reviewed and approved by the University of Houston Institutional Review Board (IRB). You may also talk to them at (713) 743-9204 or cphs@central.uh.edu if:

	Your questions, concerns, or complaints are not being answered by the research team.
	You cannot reach the research team.
	You want to talk to someone besides the research team.
	You have questions about your rights as a research subject.
	You want to get information or provide input about this research.

*Your Rights as a Research Participant:*

Your participation in this online survey is completely voluntary. You may refuse to participate, refuse to answer particular questions, and/or stop participating at any time and for any reason.

***By clicking on the Agree button below, you agree to participate in this online panel. You are free to withdraw from this panel at any time.***

- Agree  (1) 

- I do not wish to be a part of this study.  (2) 

Skip To: End of Block If Assessing Determinants of Opioid Risk Mitigation Strategies Among Physicians and Patients Investi... = I do not wish to be a part of this study.

End of Block: Default Question Block

Start of Block: Screening Physician

|  |
| --- |

Q32 Please enter your current age. 

________________________________________________________________

Skip To: End of Block If Condition: Please enter your current a... Is Less Than 18. Skip To: End of Block.

Q39 Are you currently licensed to practice medicine as a physician in the United States?

- Yes  (1) 

- No  (2) 

Skip To: End of Block If Are you currently licensed to practice medicine as a physician in the United States? = No

Q40 Have you ever prescribed any of the following opioid medications?

 

- Yes  (1) 

- No  (4) 

Skip To: End of Block If Have you ever prescribed any of the following opioid medications?   = No

Q42 Have you prescribed any of these medications ***within the past year***?

 

 

- Yes  (1) 

- No  (2) 

Skip To: End of Block If Have you prescribed any of these medications within the past year?     = No

End of Block: Screening Physician

Start of Block: Block 1

Q3 
In the next few pages, you will be presented with some introductory information about various opioid risk mitigation strategies recommended by the Centers for Disease Control and Prevention (CDC) to prevent opioid abuse and misuse. First, you will read a short description for each of nine opioid risk mitigation strategies. Then you provide your rating for three questions and provide the justification for your ratings in an open-ended question.

End of Block: Block 1

Start of Block: RMS1

Q48 **Risk Mitigation Strategy 1: Documentation of Treatment Goals**

The CDC recommends that before starting opioid therapy for chronic pain, clinicians should establish treatment goals with all patients, including realistic goals for pain and function. Clinicians should also consider how to discontinue opioid therapy if benefits do not outweigh risks. These treatment goals should be documented and agreed between patient and physician.

After reading the description of Risk Mitigation Strategy 1, please rate the following:

Q4 How would you rate your level of comfort for opioid risk mitigation strategy 1?

|  |  |  |  |  |  |  |  |
| --- | --- | --- | --- | --- | --- | --- | --- |
| 1 (1) | <span style="font-size:16px;">Extremely Uncomfortable</span> (1) | <span style="font-size:16px;">Uncomfortable</span> (2) | <span style="font-size:16px;">Somewhat Uncomfortable</span> (3) | <span style="font-size:16px;">Neutral</span> (4) | <span style="font-size:16px;">Somewhat Comfortable</span> (5) | <span style="font-size:16px;">Comfortable</span> (6) | <span style="font-size:16px;">Extremely Comfortable</span> (7) |

Q46 How would you rate the effectiveness of opioid risk mitigation strategy 1?

|  |  |  |  |  |  |  |  |
| --- | --- | --- | --- | --- | --- | --- | --- |
| 2 (2) | <span style="font-size:16px;">Extremely Ineffective</span> (1) | <span style="font-size:16px;">Ineffective</span> (2) | <span style="font-size:16px;">Somewhat Ineffective</span> (3) | <span style="font-size:16px;">Neutral</span> (4) | <span style="font-size:16px;">Somewhat Effective</span> (5) | <span style="font-size:16px;">Effective</span> (6) | <span style="font-size:16px;">Extremely Effective</span> (7) |

Q47 How would you rate the ease of implementation for opioid risk mitigation strategy 1?

|  |  |  |  |  |  |  |  |
| --- | --- | --- | --- | --- | --- | --- | --- |
| 3 (3) | <span style="font-size:16px;">Extremely Difficult</span> (1) | <span style="font-size:16px;">Difficult</span> (2) | <span style="font-size:16px;">Somewhat Difficult</span> (3) | <span style="font-size:16px;">Neutral</span> (4) | <span style="font-size:16px;">Somewhat Easy</span> (5) | <span style="font-size:16px;">Easy</span> (6) | <span style="font-size:16px;">Extremely Easy</span> (7) |

Q13 Please explain the justification for your ratings.

________________________________________________________________

________________________________________________________________

________________________________________________________________

________________________________________________________________

________________________________________________________________

End of Block: RMS1

Start of Block: RMS2

Q50 **Risk Mitigation Strategy 2: Screening for Risk of Opioid Use Disorder**

 

The concern for addiction risk is shared by physicians, patients, and their families. Some patients with chronic pain may suffer from a prior history of addiction. Alternatively, even patients without any history of substance use issues may go on to develop aberrant drug-related behaviors (ADRB). Therefore, it is important to screen patients for their risk of developing an opioid use disorder (OUD) before initiating opioid therapy. In order to screen patients for their risk of developing an OUD, there are many different tools. Some examples are: 

 

 Opioid Risk Tool (ORT) – This is a 5-question screening tool designed for use in adults to assess for the risk for opioid abuse or ADRB.
 Screener and Opioid Assessment for Patients with Pain-Revised (SOAPP®-R) – This is a 24-question screening tool that is designed to predict ADRB prior to the initiation of long-term opioid therapy.

 

After reading the description of Risk Mitigation Strategy 2, please rate the following:

Q51 How would you rate your level of comfort for opioid risk mitigation strategy 2?

|  |  |  |  |  |  |  |  |
| --- | --- | --- | --- | --- | --- | --- | --- |
| 1 (1) | <span style="font-size:16px;">Extremely Uncomfortable</span> (1) | <span style="font-size:16px;">Uncomfortable</span> (2) | <span style="font-size:16px;">Somewhat Uncomfortable</span> (3) | <span style="font-size:16px;">Neutral</span> (4) | <span style="font-size:16px;">Somewhat Comfortable</span> (5) | <span style="font-size:16px;">Comfortable</span> (6) | <span style="font-size:16px;">Extremely Comfortable</span> (7) |

Q52 How would you rate the effectiveness of opioid risk mitigation strategy 2?

|  |  |  |  |  |  |  |  |
| --- | --- | --- | --- | --- | --- | --- | --- |
| 2 (2) | <span style="font-size:16px;">Extremely Ineffective</span> (1) | <span style="font-size:16px;">Ineffective</span> (2) | <span style="font-size:16px;">Somewhat Ineffective</span> (3) | <span style="font-size:16px;">Neutral</span> (4) | <span style="font-size:16px;">Somewhat Effective</span> (5) | <span style="font-size:16px;">Effective</span> (6) | <span style="font-size:16px;">Extremely Effective</span> (7) |

Q53 How would you rate the ease of implementation for opioid risk mitigation strategy 2?

|  |  |  |  |  |  |  |  |
| --- | --- | --- | --- | --- | --- | --- | --- |
| 3 (3) | <span style="font-size:16px;">Extremely Difficult</span> (1) | <span style="font-size:16px;">Difficult</span> (2) | <span style="font-size:16px;">Somewhat Difficult</span> (3) | <span style="font-size:16px;">Neutral</span> (4) | <span style="font-size:16px;">Somewhat Easy</span> (5) | <span style="font-size:16px;">Easy</span> (6) | <span style="font-size:16px;">Extremely Easy</span> (7) |

Q54 Please explain the justification for your ratings.

________________________________________________________________

________________________________________________________________

________________________________________________________________

________________________________________________________________

________________________________________________________________

End of Block: RMS2

Start of Block: RMS3

Q55 **Risk Mitigation Strategy 3: Limiting the Day Supply of an Opioid Prescription**

The CDC recognizes that long-term opioid use often begins with the treatment of acute pain. Therefore, in order to prevent long-term reliance on opioid medications, they recommend that clinicians limit the day supply of opioid prescriptions to three days or less for the treatment of acute pain. They also state that prescriptions for more than seven days will rarely be needed and should be avoided.

After reading the description of Risk Mitigation Strategy 3, please rate the following:

Q56 How would you rate your level of comfort for opioid risk mitigation strategy 3?

|  |  |  |  |  |  |  |  |
| --- | --- | --- | --- | --- | --- | --- | --- |
| 1 (1) | <span style="font-size:16px;">Extremely Uncomfortable</span> (1) | <span style="font-size:16px;">Uncomfortable</span> (2) | <span style="font-size:16px;">Somewhat Uncomfortable</span> (3) | <span style="font-size:16px;">Neutral</span> (4) | <span style="font-size:16px;">Somewhat Comfortable</span> (5) | <span style="font-size:16px;">Comfortable</span> (6) | <span style="font-size:16px;">Extremely Comfortable</span> (7) |

Q57 How would you rate the effectiveness of opioid risk mitigation strategy 3?

|  |  |  |  |  |  |  |  |
| --- | --- | --- | --- | --- | --- | --- | --- |
| 2 (2) | <span style="font-size:16px;">Extremely Ineffective</span> (1) | <span style="font-size:16px;">Ineffective</span> (2) | <span style="font-size:16px;">Somewhat Ineffective</span> (3) | <span style="font-size:16px;">Neutral</span> (4) | <span style="font-size:16px;">Somewhat Effective</span> (5) | <span style="font-size:16px;">Effective</span> (6) | <span style="font-size:16px;">Extremely Effective</span> (7) |

Q58 How would you rate the ease of implementation for opioid risk mitigation strategy 3?

|  |  |  |  |  |  |  |  |
| --- | --- | --- | --- | --- | --- | --- | --- |
| 3 (3) | <span style="font-size:16px;">Extremely Difficult</span> (1) | <span style="font-size:16px;">Difficult</span> (2) | <span style="font-size:16px;">Somewhat Difficult</span> (3) | <span style="font-size:16px;">Neutral</span> (4) | <span style="font-size:16px;">Somewhat Easy</span> (5) | <span style="font-size:16px;">Easy</span> (6) | <span style="font-size:16px;">Extremely Easy</span> (7) |

Q59 Please explain the justification for your ratings.

________________________________________________________________

________________________________________________________________

________________________________________________________________

________________________________________________________________

________________________________________________________________

End of Block: RMS3

Start of Block: RMS4

Q60 
**Risk Mitigation Strategy 4: Limiting the Dosage of an Opioid Prescription**

 
One way to mitigate the potential for dependence is by prescribing lower dosages of opioids. Higher doses of opioids do not always translate to more pain relief due to tolerance. Therefore, placing a maximum dosage cap may prevent opioid misuse.

 

After reading the description of Risk Mitigation Strategy 4, please rate the following:

Q61 How would you rate your level of comfort for opioid risk mitigation strategy 4?

|  |  |  |  |  |  |  |  |
| --- | --- | --- | --- | --- | --- | --- | --- |
| 1 (1) | <span style="font-size:16px;">Extremely Uncomfortable</span> (1) | <span style="font-size:16px;">Uncomfortable</span> (2) | <span style="font-size:16px;">Somewhat Uncomfortable</span> (3) | <span style="font-size:16px;">Neutral</span> (4) | <span style="font-size:16px;">Somewhat Comfortable</span> (5) | <span style="font-size:16px;">Comfortable</span> (6) | <span style="font-size:16px;">Extremely Comfortable</span> (7) |

Q62 How would you rate the effectiveness of opioid risk mitigation strategy 4?

|  |  |  |  |  |  |  |  |
| --- | --- | --- | --- | --- | --- | --- | --- |
| 2 (2) | <span style="font-size:16px;">Extremely Ineffective</span> (1) | <span style="font-size:16px;">Ineffective</span> (2) | <span style="font-size:16px;">Somewhat Ineffective</span> (3) | <span style="font-size:16px;">Neutral</span> (4) | <span style="font-size:16px;">Somewhat Effective</span> (5) | <span style="font-size:16px;">Effective</span> (6) | <span style="font-size:16px;">Extremely Effective</span> (7) |

Q63 How would you rate the ease of implementation for opioid risk mitigation strategy 4?

|  |  |  |  |  |  |  |  |
| --- | --- | --- | --- | --- | --- | --- | --- |
| 3 (3) | <span style="font-size:16px;">Extremely Difficult</span> (1) | <span style="font-size:16px;">Difficult</span> (2) | <span style="font-size:16px;">Somewhat Difficult</span> (3) | <span style="font-size:16px;">Neutral</span> (4) | <span style="font-size:16px;">Somewhat Easy</span> (5) | <span style="font-size:16px;">Easy</span> (6) | <span style="font-size:16px;">Extremely Easy</span> (7) |

Q64 Please explain the justification for your ratings.

________________________________________________________________

________________________________________________________________

________________________________________________________________

________________________________________________________________

________________________________________________________________

End of Block: RMS4

Start of Block: RMS5

Q65 
**Risk Mitigation Strategy 5: Reviewing the PDMP Prior to Prescribing an Opioid Medication**

 
Prescription Drug Monitoring Programs (PDMPs) are state-maintained databases that keep track of controlled substance prescribing and dispensing within the state and other partnered states using the same software platform. They can be used to identify a patient’s history of controlled substances, as well as concomitant use of controlled substances.

 

After reading the description of Risk Mitigation Strategy 5, please rate the following:

Q66 How would you rate your level of comfort for opioid risk mitigation strategy 5?

|  |  |  |  |  |  |  |  |
| --- | --- | --- | --- | --- | --- | --- | --- |
| 1 (1) | <span style="font-size:16px;">Extremely Uncomfortable</span> (1) | <span style="font-size:16px;">Uncomfortable</span> (2) | <span style="font-size:16px;">Somewhat Uncomfortable</span> (3) | <span style="font-size:16px;">Neutral</span> (4) | <span style="font-size:16px;">Somewhat Comfortable</span> (5) | <span style="font-size:16px;">Comfortable</span> (6) | <span style="font-size:16px;">Extremely Comfortable</span> (7) |

Q67 How would you rate the effectiveness of opioid risk mitigation strategy 5?

|  |  |  |  |  |  |  |  |
| --- | --- | --- | --- | --- | --- | --- | --- |
| 2 (2) | <span style="font-size:16px;">Extremely Ineffective</span> (1) | <span style="font-size:16px;">Ineffective</span> (2) | <span style="font-size:16px;">Somewhat Ineffective</span> (3) | <span style="font-size:16px;">Neutral</span> (4) | <span style="font-size:16px;">Somewhat Effective</span> (5) | <span style="font-size:16px;">Effective</span> (6) | <span style="font-size:16px;">Extremely Effective</span> (7) |

Q68 How would you rate the ease of implementation for opioid risk mitigation strategy 5?

|  |  |  |  |  |  |  |  |
| --- | --- | --- | --- | --- | --- | --- | --- |
| 3 (3) | <span style="font-size:16px;">Extremely Difficult</span> (1) | <span style="font-size:16px;">Difficult</span> (2) | <span style="font-size:16px;">Somewhat Difficult</span> (3) | <span style="font-size:16px;">Neutral</span> (4) | <span style="font-size:16px;">Somewhat Easy</span> (5) | <span style="font-size:16px;">Easy</span> (6) | <span style="font-size:16px;">Extremely Easy</span> (7) |

Q69 Please explain the justification for your ratings.

________________________________________________________________

________________________________________________________________

________________________________________________________________

________________________________________________________________

________________________________________________________________

End of Block: RMS5

Start of Block: RMS6

Q70 **Risk Mitigation Strategy 6: Limiting Concurrent Benzodiazepine Prescriptions**  

Patients who have taken benzodiazepine and opioid medication concurrently have been known to experience adverse events ranging from respiratory depression to overdose. Recognizing the risk of co-prescribing, in 2016 the US Food and Drug Administration issued a warning to patients and clinicians about the potential risks of combined opioid and benzodiazepine use. However, the panel that wrote the guidelines noted that there may be clinically appropriate situations where the drugs may be used together, giving the example of a person taking a stable low dose of a benzodiazepine who experiences acute pain.  

After reading the description of Risk Mitigation Strategy 6, please rate the following:

Q71 How would you rate your level of comfort for opioid risk mitigation strategy 6?

|  |  |  |  |  |  |  |  |
| --- | --- | --- | --- | --- | --- | --- | --- |
| 1 (1) | <span style="font-size:16px;">Extremely Uncomfortable</span> (1) | <span style="font-size:16px;">Uncomfortable</span> (2) | <span style="font-size:16px;">Somewhat Uncomfortable</span> (3) | <span style="font-size:16px;">Neutral</span> (4) | <span style="font-size:16px;">Somewhat Comfortable</span> (5) | <span style="font-size:16px;">Comfortable</span> (6) | <span style="font-size:16px;">Extremely Comfortable</span> (7) |

Q72 How would you rate the effectiveness of opioid risk mitigation strategy 6?

|  |  |  |  |  |  |  |  |
| --- | --- | --- | --- | --- | --- | --- | --- |
| 2 (2) | <span style="font-size:16px;">Extremely Ineffective</span> (1) | <span style="font-size:16px;">Ineffective</span> (2) | <span style="font-size:16px;">Somewhat Ineffective</span> (3) | <span style="font-size:16px;">Neutral</span> (4) | <span style="font-size:16px;">Somewhat Effective</span> (5) | <span style="font-size:16px;">Effective</span> (6) | <span style="font-size:16px;">Extremely Effective</span> (7) |

Q73 How would you rate the ease of implementation for opioid risk mitigation strategy 6?

|  |  |  |  |  |  |  |  |
| --- | --- | --- | --- | --- | --- | --- | --- |
| 3 (3) | <span style="font-size:16px;">Extremely Difficult</span> (1) | <span style="font-size:16px;">Difficult</span> (2) | <span style="font-size:16px;">Somewhat Difficult</span> (3) | <span style="font-size:16px;">Neutral</span> (4) | <span style="font-size:16px;">Somewhat Easy</span> (5) | <span style="font-size:16px;">Easy</span> (6) | <span style="font-size:16px;">Extremely Easy</span> (7) |

Q74 Please explain the justification for your ratings.

________________________________________________________________

________________________________________________________________

________________________________________________________________

________________________________________________________________

________________________________________________________________

End of Block: RMS6

Start of Block: RMS7

Q75 **Risk Mitigation Strategy 7: Urine Drug Screening**

 

Urine drug testing, also known as urine drug screening, is a painless test that analyzes your urine for the presence of certain prescription medications. The CDC recommends urine drug screening both before the initiation of opioid therapy and at least annually during opioid therapy. 

 

After reading the description of Risk Mitigation Strategy 7, please rate the following:

 

Q76 How would you rate your level of comfort for opioid risk mitigation strategy 7?

|  |  |  |  |  |  |  |  |
| --- | --- | --- | --- | --- | --- | --- | --- |
| 1 (1) | <span style="font-size:16px;">Extremely Uncomfortable</span> (1) | <span style="font-size:16px;">Uncomfortable</span> (2) | <span style="font-size:16px;">Somewhat Uncomfortable</span> (3) | <span style="font-size:16px;">Neutral</span> (4) | <span style="font-size:16px;">Somewhat Comfortable</span> (5) | <span style="font-size:16px;">Comfortable</span> (6) | <span style="font-size:16px;">Extremely Comfortable</span> (7) |

Q77 How would you rate the effectiveness of opioid risk mitigation strategy 7?

|  |  |  |  |  |  |  |  |
| --- | --- | --- | --- | --- | --- | --- | --- |
| 2 (2) | <span style="font-size:16px;">Extremely Ineffective</span> (1) | <span style="font-size:16px;">Ineffective</span> (2) | <span style="font-size:16px;">Somewhat Ineffective</span> (3) | <span style="font-size:16px;">Neutral</span> (4) | <span style="font-size:16px;">Somewhat Effective</span> (5) | <span style="font-size:16px;">Effective</span> (6) | <span style="font-size:16px;">Extremely Effective</span> (7) |

Q78 How would you rate the ease of implementation for opioid risk mitigation strategy 7?

|  |  |  |  |  |  |  |  |
| --- | --- | --- | --- | --- | --- | --- | --- |
| 3 (3) | <span style="font-size:16px;">Extremely Difficult</span> (1) | <span style="font-size:16px;">Difficult</span> (2) | <span style="font-size:16px;">Somewhat Difficult</span> (3) | <span style="font-size:16px;">Neutral</span> (4) | <span style="font-size:16px;">Somewhat Easy</span> (5) | <span style="font-size:16px;">Easy</span> (6) | <span style="font-size:16px;">Extremely Easy</span> (7) |

Q79 Please explain the justification for your ratings.

________________________________________________________________

________________________________________________________________

________________________________________________________________

________________________________________________________________

________________________________________________________________

End of Block: RMS7

Start of Block: RMS8

Q80 **Risk Mitigation Strategy 8: Safe Drug Disposal**

One way to reduce the number of opioid-related overdoses and deaths is to ensure the proper disposal of opioid medications. This can be done through a variety of ways. The Drug Enforcement Administration (DEA) sponsors National Drug Take Back days, where central locations are set up for residents to drop off their unused or leftover medications to be disposed of safely. Another alternative is Single Use Disposal Systems (SUDS). These systems allow for patients to safely dispose of their medications from home. Physicians can play an important role in ensuring that patients are aware of the various safe disposal options available to them.

After reading the description of Risk Mitigation Strategy 8, please rate the following:

Q81 How would you rate your level of comfort for opioid risk mitigation strategy 8?

|  |  |  |  |  |  |  |  |
| --- | --- | --- | --- | --- | --- | --- | --- |
| 1 (1) | <span style="font-size:16px;">Extremely Uncomfortable</span> (1) | <span style="font-size:16px;">Uncomfortable</span> (2) | <span style="font-size:16px;">Somewhat Uncomfortable</span> (3) | <span style="font-size:16px;">Neutral</span> (4) | <span style="font-size:16px;">Somewhat Comfortable</span> (5) | <span style="font-size:16px;">Comfortable</span> (6) | <span style="font-size:16px;">Extremely Comfortable</span> (7) |

Q82 How would you rate the effectiveness of opioid risk mitigation strategy 8?

|  |  |  |  |  |  |  |  |
| --- | --- | --- | --- | --- | --- | --- | --- |
| 2 (2) | <span style="font-size:16px;">Extremely Ineffective</span> (1) | <span style="font-size:16px;">Ineffective</span> (2) | <span style="font-size:16px;">Somewhat Ineffective</span> (3) | <span style="font-size:16px;">Neutral</span> (4) | <span style="font-size:16px;">Somewhat Effective</span> (5) | <span style="font-size:16px;">Effective</span> (6) | <span style="font-size:16px;">Extremely Effective</span> (7) |

Q83 How would you rate the ease of implementation for opioid risk mitigation strategy 8?

|  |  |  |  |  |  |  |  |
| --- | --- | --- | --- | --- | --- | --- | --- |
| 3 (3) | <span style="font-size:16px;">Extremely Difficult</span> (1) | <span style="font-size:16px;">Difficult</span> (2) | <span style="font-size:16px;">Somewhat Difficult</span> (3) | <span style="font-size:16px;">Neutral</span> (4) | <span style="font-size:16px;">Somewhat Easy</span> (5) | <span style="font-size:16px;">Easy</span> (6) | <span style="font-size:16px;">Extremely Easy</span> (7) |

Q84 Please explain the justification for your ratings.

________________________________________________________________

________________________________________________________________

________________________________________________________________

________________________________________________________________

________________________________________________________________

End of Block: RMS8

Start of Block: RMS9

Q85 **Risk Mitigation Strategy 9: Referral Services**

Physicians use a variety of tools (PDMP review, urine drug testing, OUD Screening) to assess the potential of opioid dependency among their patients. If a patient is thought to be misusing or developing a dependency on their opioid medications, physicians can refer their patients to treatment. More specifically, the FDA recommends Medication Assisted Treatment (MAT) to treat OUD.

After reading the description of Risk Mitigation Strategy 9, please rate the following:

Q86 How would you rate your level of comfort for opioid risk mitigation strategy 9?

|  |  |  |  |  |  |  |  |
| --- | --- | --- | --- | --- | --- | --- | --- |
| 1 (1) | <span style="font-size:16px;">Extremely Uncomfortable</span> (1) | <span style="font-size:16px;">Uncomfortable</span> (2) | <span style="font-size:16px;">Somewhat Uncomfortable</span> (3) | <span style="font-size:16px;">Neutral</span> (4) | <span style="font-size:16px;">Somewhat Comfortable</span> (5) | <span style="font-size:16px;">Comfortable</span> (6) | <span style="font-size:16px;">Extremely Comfortable</span> (7) |

Q87 How would you rate the effectiveness of opioid risk mitigation strategy 9?

|  |  |  |  |  |  |  |  |
| --- | --- | --- | --- | --- | --- | --- | --- |
| 2 (2) | <span style="font-size:16px;">Extremely Ineffective</span> (1) | <span style="font-size:16px;">Ineffective</span> (2) | <span style="font-size:16px;">Somewhat Ineffective</span> (3) | <span style="font-size:16px;">Neutral</span> (4) | <span style="font-size:16px;">Somewhat Effective</span> (5) | <span style="font-size:16px;">Effective</span> (6) | <span style="font-size:16px;">Extremely Effective</span> (7) |

Q88 How would you rate the ease of implementation for opioid risk mitigation strategy 9?

|  |  |  |  |  |  |  |  |
| --- | --- | --- | --- | --- | --- | --- | --- |
| 3 (3) | <span style="font-size:16px;">Extremely Difficult</span> (1) | <span style="font-size:16px;">Difficult</span> (2) | <span style="font-size:16px;">Somewhat Difficult</span> (3) | <span style="font-size:16px;">Neutral</span> (4) | <span style="font-size:16px;">Somewhat Easy</span> (5) | <span style="font-size:16px;">Easy</span> (6) | <span style="font-size:16px;">Extremely Easy</span> (7) |

Q89 Please explain the justification for your ratings.

________________________________________________________________

________________________________________________________________

________________________________________________________________

________________________________________________________________

________________________________________________________________

End of Block: RMS9

Start of Block: Rank Order

|  |
| --- |

Q22 In the previous sections, you have rated nine separate risk mitigation strategies on their acceptability, effectiveness, and ease of implementation. In this section, please rank the risk mitigation strategies in order of your preference.

______ Documentation of Treatment Goals (1)

______ Screening for OUD Risk (2)

______ Limiting the day supply of an opioid prescription (3)

______ Limiting the dosage of an opioid prescription (4)

______ Reviewing the PDMP prior to prescribing an opioid medication (5)

______ Limiting Concurrent Benzodiazepine Prescriptions (6)

______ Urine Drug Screening (7)

______ Safe Drug Disposal (8)

______ Referral Services (9)

End of Block: Rank Order

Start of Block: Demographic Questions - Physician

Q24 Which of the following best describes your racial/ethnic background?

- White  (2) 

- Hispanic or Latino  (3) 

- Black or African American  (6) 

- Native American or American Indian  (7) 

- Asian or other Pacific Islander  (8) 

- Other, Please Specify  (9) __________________________________________________

Q33 What is your sex?

- Male  (1) 

- Female  (2) 

Q23 Please enter your practice specialty. 

________________________________________________________________

Q38 Have you ever received an opioid medication?

- Yes  (1) 

- No  (2) 

|  |
| --- |

Q90 What is your current STATE of residence?

________________________________________________________________

Q91 What is your STATE of practice?

________________________________________________________________

End of Block: Demographic Questions - Physician
